# Ofloxacin, paracetamol and cefixime induced Stevens-Johnson syndrome - toxic epidermal necrolysis in an adult female patient: a case report

**DOI:** 10.1186/s40780-025-00488-5

**Published:** 2025-10-31

**Authors:** Margi R. Desai, Harshita Jaiswal, Mrudangsinh M. Rathod, Sakshi Vijayvargi

**Affiliations:** 1 Department of Pharmacy Practice, Parul Institute of Pharmacy, Parul University, Vadodara, Gujarat India; 2 Department of Pharmacy Practice, School of Pharmacy, Parul University, P.O. limda, Tal. Waghodiya,, Vadodara, 391760 Gujarat India; 3 Department of Pharmacy Practice, Parul Institute of Pharmacy, Parul University, Vadodara,, Gujarat India; 4 CBCC Global Research, Ahmedabad, Gujarat India

**Keywords:** Stevens-Johnson syndrome, Toxic epidermal necrolysis, Drug-induced hypersensitivity, Polypharmacy, Early diagnosis, Pharmacovigilance, Case report, Mucocutaneous reaction, Individualized treatment

## Abstract

**Background:**

Stevens-Johnson Syndrome (SJS) and Toxic Epidermal Necrolysis (TEN) represent life-threatening mucocutaneous reactions, predominantly triggered by medications. This case report presents a rare instance of SJS-TEN overlap in a young Indian female precipitated by the combined use of fluoroquinolone (ofloxacin), cephalosporin (cefixime), and paracetamol—an uncommon drug triad not widely reported in existing literature. The case highlights the importance of early diagnosis, thorough drug history evaluation, and the challenges of managing polypharmacy-induced severe cutaneous adverse reactions (SCARs).

**Case presentation:**

A 29-year-old Indian female developed widespread dusky purpuric plaques, mucosal erosions (oral, genital, conjunctival, nasal), and bullae six days after local consultation with ofloxacin, cefixime, paracetamol, and other symptomatic agents. She presented to the emergency department with painful vesiculobullous eruptions involving > 10% body surface area, mucosal ulcerations, eye involvement with crusting, and systemic symptoms including fever, vomiting, and urinary discomfort. Laboratory investigations revealed anemia, elevated RDW, and positive ketones in urine. Diagnosis of SJS-TEN overlap was made clinically. The patient was managed with a multi-disciplinary approach involving systemic corticosteroids (IV dexamethasone), hydration, antibiotics (azithromycin), antihistamines, electrolyte balance, topical agents, ophthalmic care, and pain management. The extensive yet individualized treatment regimen reflected a robust pharmacovigilance response to avoid further drug-induced complications. Improvement was noted with complete re-epithelialization and symptomatic resolution over two weeks.

**Conclusion:**

This case highlights the necessity for clinicians to maintain high suspicion for SCARs in patients presenting with mucocutaneous symptoms and recent drug exposure—even with commonly used medications not frequently associated with SJS-TEN. The unique presentation involving synchronous ocular, nasal, oral, and genital erosions alongside use of a rare drug combination strengthens the need for early recognition, comprehensive clinical assessment, and cautious prescription practices. Individualized treatment and close monitoring are crucial in preventing mortality and minimizing complications. This case underscores the importance of pharmacovigilance and personalized care in managing drug-induced hypersensitivity reactions, especially in resource-limited or polypharmacy scenarios.

## Background

SJS is a type IV (subtype C) hypersensitivity reaction affecting the skin and mucous membranes [1]. TEN, also called Lyell’s syndrome, affects the mucous membranes, causing skin and mucosal shedding due to cell death. It is usually triggered by medications [2]. It is classified based on affected body surface area (BSA) detached: < 10% for SJS, 10–30% for SJS-TEN overlaps, and > 30% for TEN. It is more common in adults, especially those over 65, and slightly more common in females (3:2) [3].

SJS-TEN is severe T cell mediated delayed hypersensitivity reaction. The crucial event encompasses a tripartite interaction between a peptide presented by a major histocompatibility complex (MHC) on an antigen-presenting cell (APC) and the T cell receptor (TCR) expressed on a CD8+ (cytotoxic) T cell. These drug-reactive T cells originate from skin-resident memory T cells (T_RM_) and/or circulating CD8 + T cells. After the initial interaction, activated CD8 + T cells promote cytokine/chemokine production and keratinocyte apoptosis via the Fas/Fas Ligand and TCR/HLA (human leukocyte antigen) pathways. Keratinocytes may also trigger apoptosis in other keratinocytes through Fas/Fas Ligand signaling. NK cells contribute to keratinocyte apoptosis through CD94/NKG2C interaction with HLA-E and release granulysin. Drug-activated monocytes may induce necroptosis via Annexin A1 and FPR1 (formal peptide receptor 1) binding. Blister fluid from SJS-TEN patients contains clonally-expanded CD8 + T cells, NK cells, monocytes, macrophages, and apoptotic mediators like TRAIL, perforin, granzyme, TNF-α, soluble FasL, and granulysin. Innate immunity plays a larger role, with neutrophils contributing to inflammation and keratinocyte necroptosis through NETosis. Genetic factors, particularly HLA alleles like HLA-B*15:02*, are linked to drug-induced SJS-TEN. Screening for HLA-B15:02 before prescribing certain anticonvulsants has reduced incidence, while HLA-B*57:01 screening has eliminated abacavir hypersensitivity. Other genetic risk factors include polymorphisms in cytochrome P450 enzymes (e.g., CYP2C9, CYP2C19) and the antigen presentation pathway. Further research is needed to refine these genetic insights for clinical use [4].

Usually, lesions are observed 4–28 days after the patient administers the implicating substance [5]. First few weeks after starting treatment is important for developing these SCARS from the medication, these SCARS include blisters on the mucosa and redness adding on with flu like symptoms - malaise, fever, rhinitis, cough and headache [6]. These are followed by inflammation and discomfort in the skin and mucous membranes, as well as other systemic involvement [7]. In next few days, skin symptoms appear quickly, starting as macular atypical target-like lesions that merge and form vesicles and bullae. These progress to full-thickness necrosis, skin detachment, and sloughing, often causing severe pain. Mucosal surfaces, including the lips, mouth, throat, respiratory and gastrointestinal tracts, and genitalia, may develop necrosis, leading to ulcers, erosions, and detachment. Ocular involvement is common in acute SJS-TEN, with complications like dry eyes, corneal inflammation, trichiasis, symblepharon, and eyelid keratinization often appearing later. Loss of smell and taste (dyssomnia and dysgeusia) may occur due to mucosal damage. Systemic involvement includes skin barrier breakdown, causing dehydration, electrolyte imbalance, hypothermia, sepsis, and organ dysfunction in the respiratory, gastrointestinal, pancreatic, and liver systems. In some cases, immune cross-reactivity with bone marrow may lead to pancytopenia [4].

Usual causative medications are anticonvulsants (lamotrigine, phenobarbital, and carbamazepine), antibiotics (sulphonamides, trimethoprim-sulfamethoxazole, penicillin, macrolides, and fluoroquinolones), corticosteroids, acetaminophen, and antiretrovirals. Recent studies have shown that SJS-TEN growth is linked to anticancer medications (daunorubicin, alpelisib, fulvestrant, and enzalutamide); the use of an elastomeric pump for the treating late-stage ovarian cancer patient, most likely as a result of Ondansetron; armodafinil and modafinil; chlordiazepoxide; pirfenidone; or following a sulfadoxine–pyrimethamine overdose [4].

Critical clinical indicators during initial evaluation include skin pain, prodromal symptoms, mucositis, and a rapidly spreading rash. A comprehensive skin examination is essential, accompanied by consultations with ophthalmology, urology, and gynaecology for mucosal evaluation [8]. Initial laboratory investigations should include a complete blood count, a comprehensive metabolic panel to assess electrolyte balance, and arterial blood gas analysis to evaluate respiratory status [9]. As defined in the introduction, the clinical differentiation between SJS and TEN is based on body surface area involvement specifically of detached or detachable skin. Histologic features show full-thickness epidermal necrosis, keratinocyte apoptosis, basal vacuole change, subepidermal bullae, subepidermal clefting, mild T cell infiltrate. Drug-induced SJS-TEN may show a dermal infiltrate with high eosinophils or neutrophils. TEN may show a denser dermal mononuclear infiltrate compared to SJS [10]. Differential diagnosis of SJS-TEN involves distinguishing it from Erythema Multiforme Major (EMM), which can be identified by the appearance of targetoid lesions and through immunohistochemical tests for markers like granulysin, perforin, granzyme B, CD4, and Treg, as well as autoimmune bullous diseases, which can be ruled out using direct immunofluorescence (DIF) and ELISA. Biomarkers such as granulysin, which is highly expressed in blister fluid and serum, correlate with BSA involvement and have 80% sensitivity and 95.7% specificity for early diagnosis, with serum granulysin potentially predicting SJS-TEN 2–4 days before skin detachment. HLA testing is crucial in risk stratification, helping to avoid drugs associated with SJS-TEN, and its predictive value varies by ethnicity [4]. Rarely is a biopsy required to confirm the diagnosis. For Severity and Mortality Assessment, there are various scales; SCORTEN: A validated tool using 7 clinical indicators to predict mortality risk (age > 40, active cancer, heart rate > 120, BUN > 28, >10% BSA detachment, bicarbonate < 20, glucose > 250). It should be calculated on day 1 and 3 of hospitalization [10]. Re-SCORTEN: A revised version of SCORTEN adding the red blood cell distribution width to hemoglobin ratio (RHR), improving prognostic accuracy for mortality [10]. ABCD-10: Uses 5 indicators (age > 50, >10% BSA detachment, bicarbonate < 20, cancer, prior dialysis) to predict severity and mortality but is less accurate than SCORTEN. CRISTEN: A clinical risk model using 10 parameters (e.g., age > 65, BSA > 10%, active cancer, mucosal damage, recent corticosteroid use) to predict severity without needing laboratory tests [4].

Supportive care for SJS-TEN involves withdrawing the offending drug, hospitalization, and multidisciplinary care due to potential multi-organ involvement. Critical interventions include wound, oral, ocular, and genitourinary care, along with pain, fluid, and electrolyte management, stress ulcer prophylaxis, and nutrition support. Prevention of sepsis is crucial as it is the primary cause of mortality. Aseptic conditions and careful septic handling are necessary [11]. Systemic treatment for SJS-TEN includes cyclosporine (3–5 mg/kg/d for 2 weeks), etanercept (25–50 mg twice weekly until re-epithelialization), corticosteroids (dexamethasone 1.5 mg/kg/day for 3 days, or prednisolone 60–250 mg/day for 2–12 days), and intravenous immunoglobulin (IVIG) (4 g/kg over 3 days). Cyclosporine shows promise but can be nephrotoxic. Corticosteroids improve outcomes if given early, though they may increase infection risk. IVIG, combined with corticosteroids, shows some benefit but limited effectiveness and risks, especially in renal impairment. Etanercept, a TNF-α inhibitor, has shown superior benefits in re-epithelialization and mortality, especially in combination with corticosteroids. Treatment must be adjusted for special populations, such as pregnant patients or children [4].

## Case presentation

A 29-year-old Indian female patient with no chronic illness history such as diabetes, hypertension and autoimmune disorder etc. also no prior history of drug allergies or other hypersensitivity episodes. 6 days prior to admission, the patient developed body ache and joint pain, for which she was prescribed with tab. ofloxacin 200 mg, tab. paracetamol 500 mg, tab. cefixime 200 mg, tab. tussin-DMR, tab. ORAFAST PO at a local consultation, after which following symptoms appear oral and genital lesions which gradually progressed to skin surface and blisters around eyes. After 6 days she came to emergency department with chief complain of multiple skin lesions all over body, multiple raw areas over lips and in mouth (mouth ulcers), unable to open eyes, blackening of skin, painful fluid filled dusky erythematous purpura vesicles over bilateral upper limbs and thighs spreading towards abdomen, back, chest, face and neck, pain over joints, nausea and vomiting (3–4 episodes), decreased urine output along with burning micturition, mild fever.

### Vital examination

On arrival patient was conscious, well-oriented with febrile condition (100.2° F).

### Cutaneous examination

Examination on day 0 for respective parts of body revealed: (1) Head & Neck: crusted plaques, erosion and peeling of skin presented over both eyes [Fig. 1 (a)]. Multiple well-defined dusky purpuric plaques over face present. Little erosion over lips present [Fig. 1 (b)], (2) Chest & Abdomen: multiple dusky purpuric plaques over chest and abdomen present. Few areas with peeling of skin present [Fig. 2], (3) Lower Limb: multiple dusky purpuric plaques over thighs & lower leg present [Fig. 3 (a)], (4) Upper Limb: multiple blisters over right arm present. Multiple dusky purpuric patches over both arms and forearms [Fig. 3 (b)]. (5) Back: single well-defined erosion & peeling of skin with multiple erythrematous and dusky patches over back. Multiple bullae over back present [Fig. 4], (6) Oral mucosa: multiple erosion over palate and whitish plaques over tongue, (7) Conjunctiva: redness present, (8) Nasal: crusting present (9) Genital: erosion present.

### Laboratory investigations

In laboratory investigations complete blood count and urine analysis were performed. Detailed complete blood count reports of different dates and urine analysis report mentioned in table no. 1 and 2.

**Table 1:** Complete blood count reports of the patient on different dates

Test Name	26 Dec 2024 (Day 0)	30 Dec 2024 (Day 4)	31 Dec 2024 (Day 5)	03 Jan 2025 (Day 8)	08 Jan 2025 (Day 13)	09 Jan 2025 (Day 14)	Biological Reference
Hemoglobin	8.1	9.0	9.9	9.0	9.0	9.1	**12–15 g/dl**
Red Blood Cell Count	3.74	4.17	4.57	4.22	4.15	4.20	**3.8–4.8 10^12/L**
Hematocrit	25.4	29.2	32.4	29.4	28.5	29.0	**40–54%**
MCV	67.8	70.2	70.8	69.8	68.6	69.0	**83–101fl**
RDW	16.9	17.2	17.3	16.8	17.0	17.2	**11.5–14%**
Neutrophils	49	45	45	48	73	65	**50–62%**
Monocyte	13	12	13	14	5	4	**0–10%**
Platelet Count	356,000	562,000	545,000	630,000	639,000	555,000	**150,000–450,000 /Micro Litre**

**Table 2:** Urine analysis reports of the patient

URINE ANALYSIS		
Test Name	26 Dec 2024 (Day 0)	Biological Reference
Appearance	Hazy	**-**
Protein	Absent	**-**
Glucose	Absent	**-**
Urobilinogen	Absent	**-**
Bilirubin.	Absent	**-**
Ketone.	3+	**-**
Blood	Trace	**-**
Nitrite	Absent	**-**
Pus Cells.	Occasional	**0–10 /HPF**
RBCs.	Absent	**0–10 /HPF**
Epithelial Cells	Occasional	**-**

### Treatment

In this case report, the date of hospital admission is considered as Day 0. Various medication including corticosteroids, antibiotics, topical cream and powder, mouth gel for ulcer, and eye drops given to the patient during hospitalisation. Detailed treatment chart given in table no. 3.

**Table 3:** Treatment given to patient

Sr. No.	Drug - Generic Name	Route	Dosage	Frequency	Indication	Duration	Start Date
1.	Inj. Normal Saline	Intravenous	500 ml	1-1-1	Hydration & Electrolyte solution	15days	Day 0
2.	Inj. Dexamethasone	Intravenous	2 cc (8 mg)	OD	Corticosteroid – treat inflammatory skin condition	Initial 5 days	Day 0
			1.5 cc (6 mg)	OD	Corticosteroid – treat inflammatory skin condition	Next 5 days	Day 6
3.	Inj. Ranitidine hydrochloride	Intravenous	25 mg (2 ml)	BD	Histamine H2 receptors – reduce amount of acid production	11 days	Day 0
4.	Inj. Azithromycin	Intravenous	500 mg	OD	Antibiotic	Initial 7 days	Day 0
5.	Inj. Pheniramine	Intravenous	25 mg	OD	H1 receptor antagonists – treat allergic symptoms	6 days	Day 0
6.	Tab. Azithromycin	Oral	500 mg	OD	Antibiotic	Later 8 days	Day 8
7.	Fusidic acid cream	Topical	NA	BD	Topical antibiotic	16 days	Day 0
8.	Clotrimazole mouth paint	Topical	(1% w/v)	BD	Antifungal	8 days	Day 8
9.	Eye drop. Prednisolone	Eye drops	2 drops	2 hrly	Corticosteroid	16 days	Day 0
10.	Eye drop. Tobramycin	Eye drops	(0.3%) (2 drops)	2 hrly	Antibiotic	16 days	Day 0
11.	Eyedrop. Carboxymethylcellulose	Eye drops	(0.5% w/v) (2 drops)	2 hrly	Treatment of Dry eyes	16 days	Day 0
12.	Lidocaine viscous gargle	Oral	15 ml	BD	Painful mouth sores	16 days	Day 0
13.	Triamcinolone Acetonide mouth gel	Topical	(0.1% w/w)	BD	Corticosteroid	15 days	Day 1
14.	Benzocaine gel	Topical	NA	BD	Reduce pain, irritation, sore throat	15 days	Day 1
15.	Saline nasal drops	Nasal	2 drops	TDS	Treat nasal passage dryness	11days	Day 6
16.	Azithromycin eye ointment	Ophthalmic	NA	BD	Antibiotic	10 days	Day 7
17.	Bacitracin + Neomycin + Polymyxin B powder	Topical	(400IU) + (3400IU) + (5000IU)	BD	Treat bacterial skin infection	8 days	Day 9
18.	Betamethasone dipropionate + Zinc sulphate scalp lotion	Topical	(0.05%w/v) + (0.5% w/v)	BD	Combination of steroid and antiseptic to treat itchy swollen skin condition	8 days	Day 9
19.	Hydroxypropylmethylcellulose eye ointment	Ophthalmic	NA	BD	Lubricant	7 days	Day 9
20.	Aloe Vera + Vitamin E/Tocopherol lotion	Topical	(10.0%W/W) + (0.5%W/W)	TDS	Moisturizer	16 days	Day 0
21.	Vaseline gauge dressing	Topical	NA	BD	Wound healing	2 days	Day 13
22.	Protein x powder with milk	Oral	1 tbsp	BD	Protein Source	1 day	Day 15

**Fig. 1:**
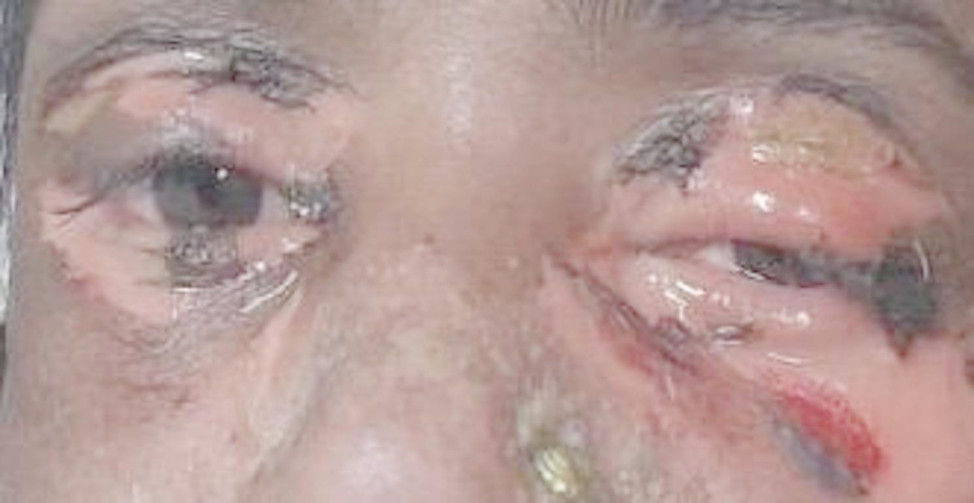
(**a**) Crusted plaques, erosions and peeling of skin presented over both eyes

**Fig. 1:**
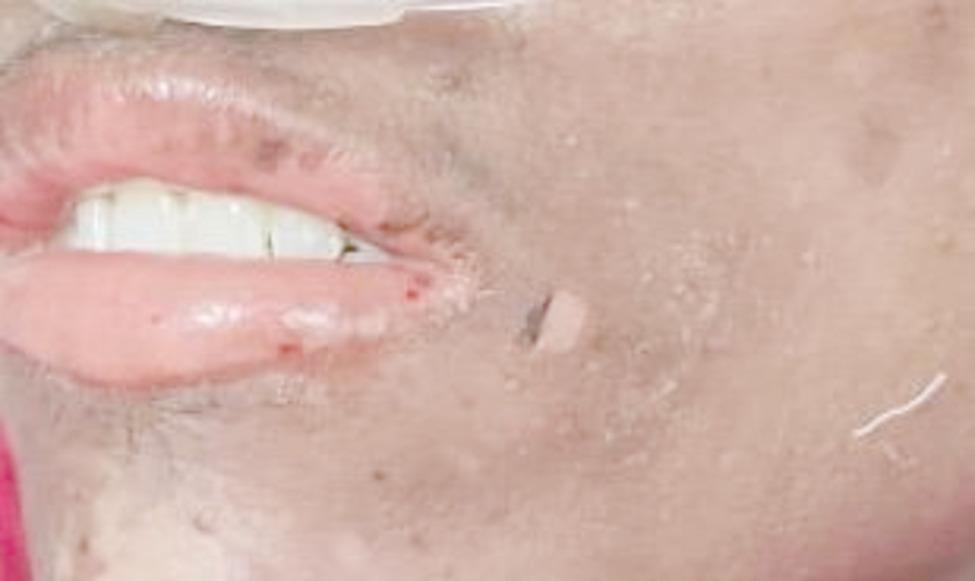
(**b**) Multiple well defined dusky purpuric plaque over face present. Little erosion over lips present

**Fig. 2:**
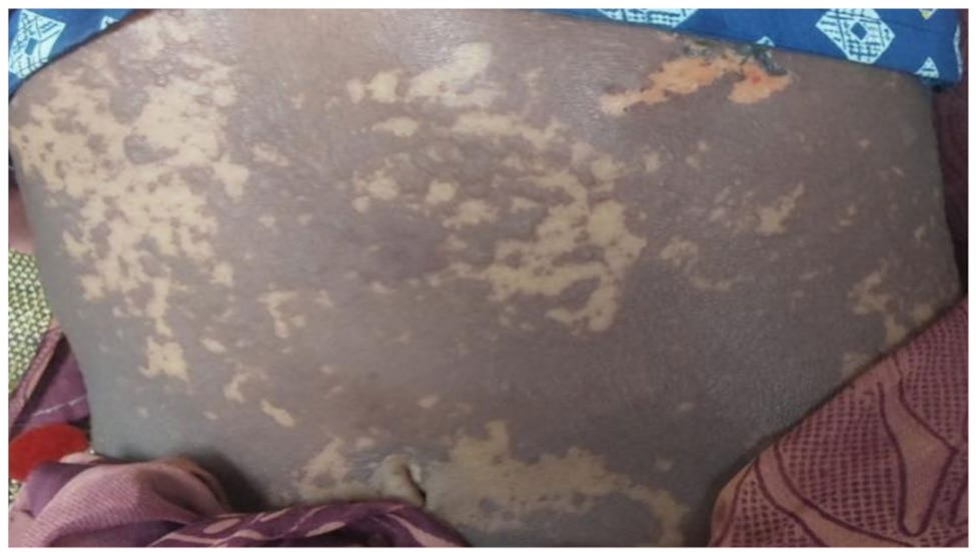
Multiple dusky purpuric plaques over chest and abdomen present. Few areas with peeling of skin present

**Fig. 3:**
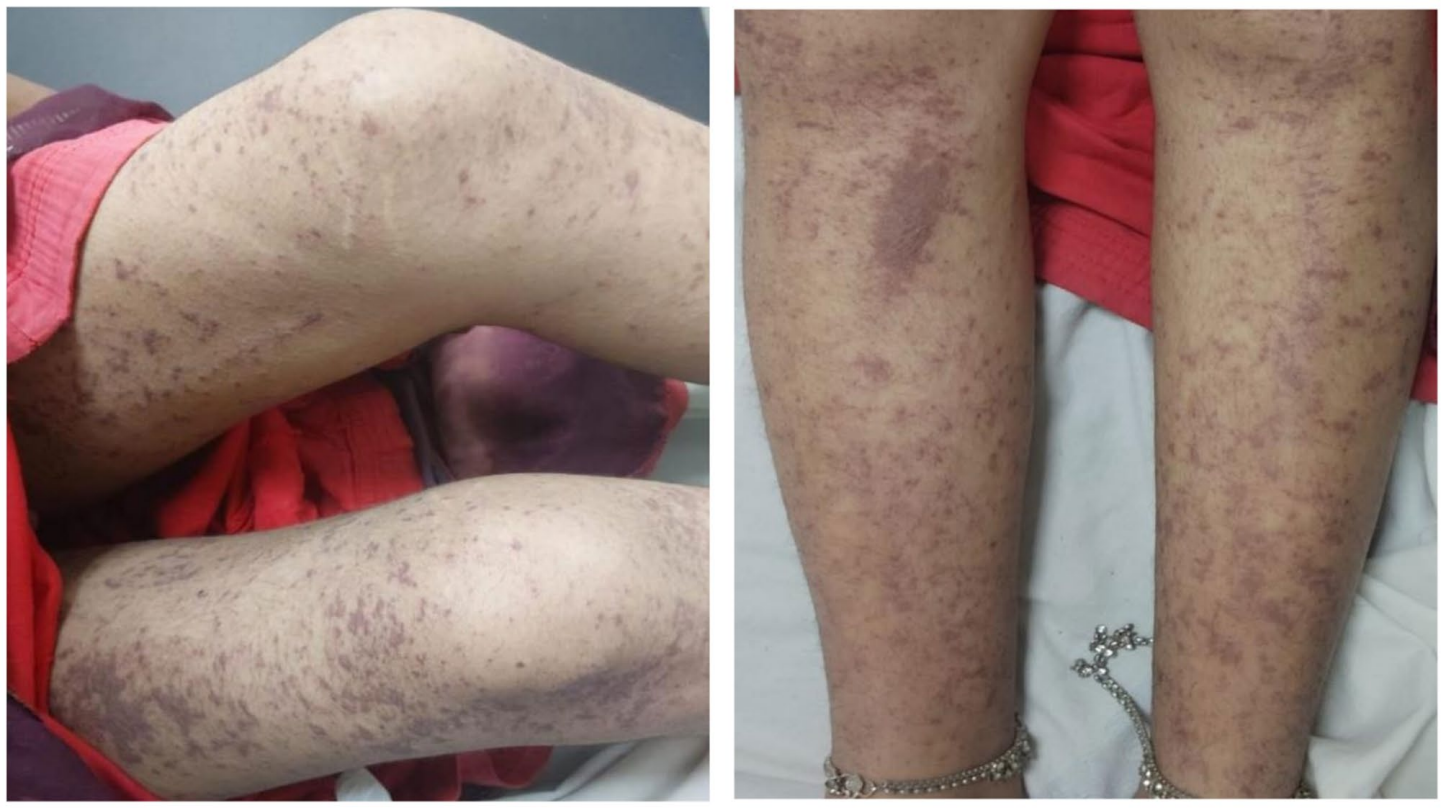
(**a**) Lower Limb: multiple dusky purpuric plaques over thighs & lower leg present

**Fig. 3:**
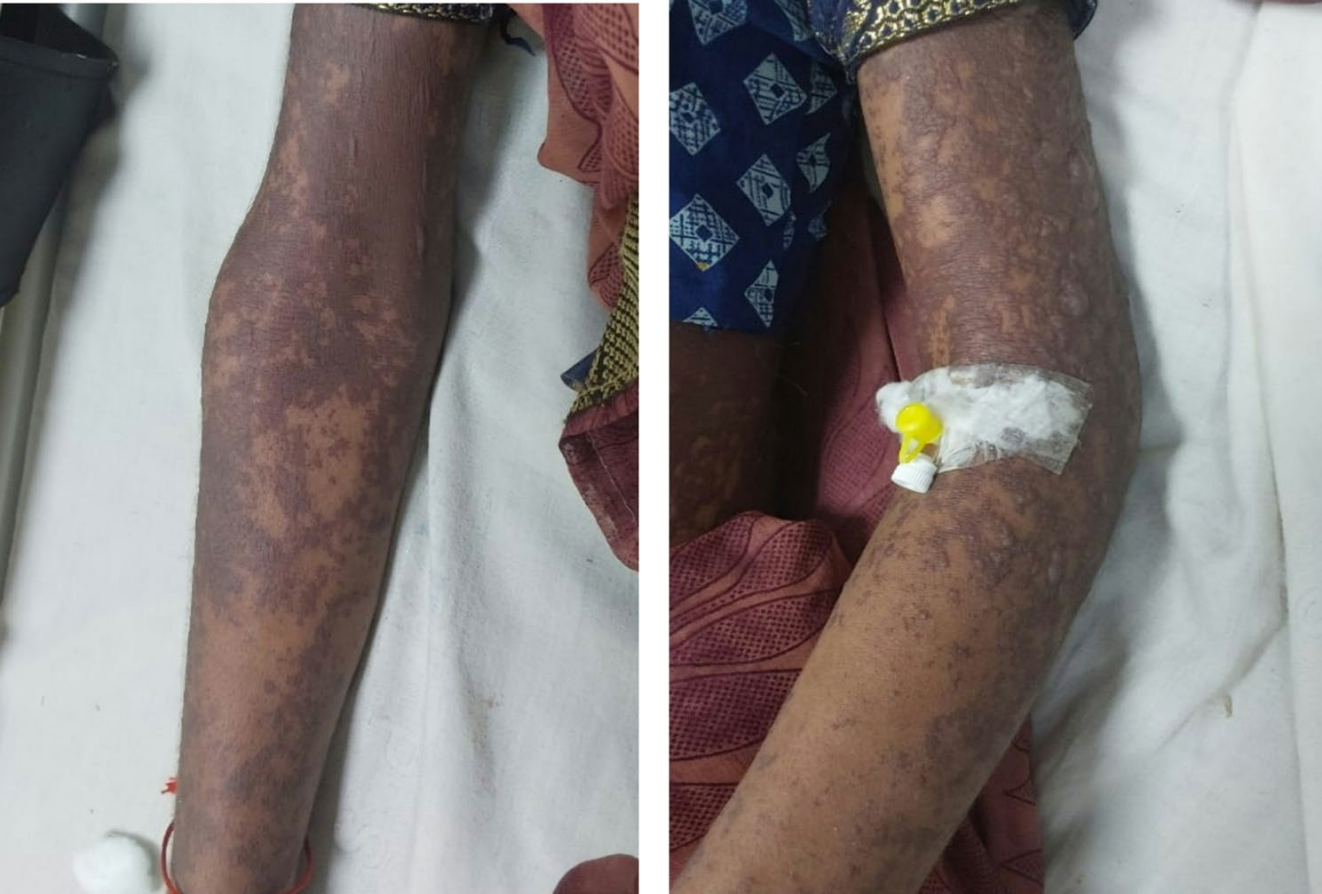
(**b**) Upper Limb: multiple blisters over right arm present. Multiple dusky purpuric patches over both arms and forearms

**Fig. 4:**
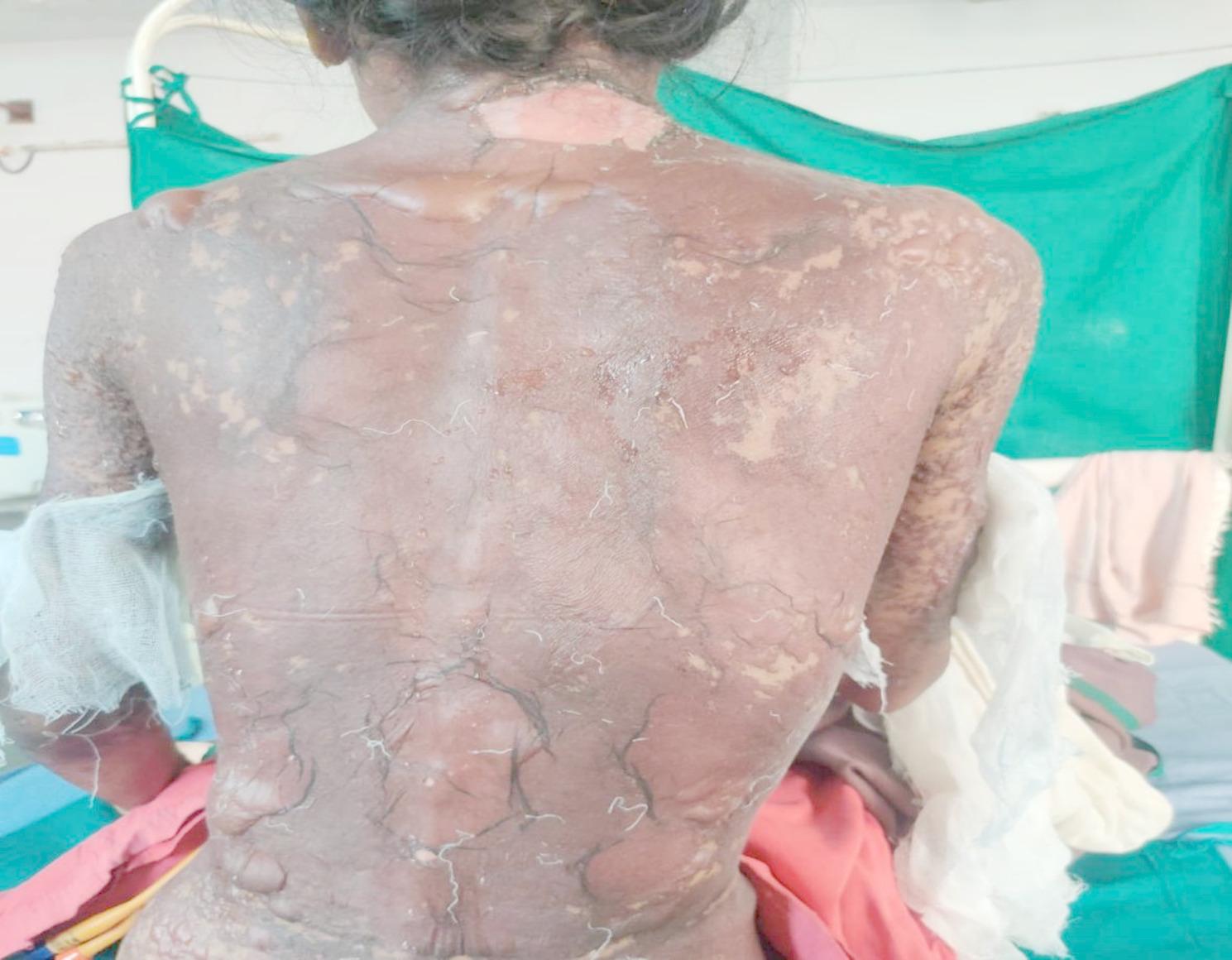
Back: single well-defined erosion & peeling of skin with multiple erythrometous and dusky patches over back. Multiple bullae over back present

### Follow-up and outcome

The patient showed gradual clinical improvement with multidisciplinary supportive care and pharmacological intervention. By the end of the second week, complete re-epithelialization of affected skin areas was observed, mucosal erosions had resolved, and systemic symptoms subsided. The patient was discharged in stable condition with advice on drug avoidance and dermatological follow-up.

### Clinical response

On Day 0 (Onset) of initial symptoms, such as high-grade fever, sore throat, burning sensation in eyes, and appearance of erythematous maculopapular rashes. On Day 1–3 there was spread of lesions to the trunk, limbs, face, and mucosal surfaces (oral, ocular, genital). Note progression to vesicles, bullae, and epidermal detachment (mention percentage of BSA involved) along with mucosal erosions and crusting. Day 4–7 Include worsening or stabilization, Nikolsky sign positivity, sloughing of skin, and onset of healing in certain areas. On Day 8 onward report re-epithelialization, reduction in erythema, and scab formation.

## Discussion

SJS-TEN is most commonly linked with drugs such as sulphonamides, anticonvulsants, allopurinol, and NSAIDs [1, 4]. In contrast, our patient developed SJS-TEN following treatment with ofloxacin and cefixime, both fluoroquinolone and cephalosporin alongside paracetamol. The typical latency period between drug exposure and symptom onset is 4–28 days [5], aligning well with the patient’s 6-day progression from drug intake to full-blown symptoms, reinforcing the diagnostic timeline established in literature. The patient’s clinical features matched classical SJS-TEN symptomatology: dusky purpuric plaques, mucosal erosions, bullae formation, conjunctivitis, genital involvement, and systemic symptoms such as fever, nausea, and joint pain. Literature widely supports these manifestations, especially ocular and mucosal involvement [1, 4, 6]. The simultaneous presentation of conjunctival, nasal, oral, and genital mucosal erosions in a single patient is reflecting the severe multisystem involvement and perhaps indicating a fulminant variant of the disease. The appearance of crusted plaques and erosions specifically over both eyes (Fig. 1a) with early conjunctival redness is significant. Ocular complications in SJS-TEN are common but often evolve later [3, 4]. Here, early onset of bilateral ocular involvement suggests that eye symptoms might, in some cases, precede other mucosal signs, challenging the typical progression pattern described in literature. The elevated RDW aligns with parameters used in Re-SCORTEN, a prognostic tool not widely implemented in routine settings, but showing superior predictive value over SCORTEN in recent studies [10]. This case supports the incorporation of such emerging indicators into early risk assessment protocols. Skin biopsy was not performed; the clinical diagnosis was evident through symptomatology and progression. The current literature supports that biopsy is rarely required in classical presentations [10]. The treatment provided aligns with current best practices, including systemic corticosteroids, antibiotics, eye care, topical agents, and nutritional support. Dexamethasone was administered for the initial 10 days, consistent with evidence suggesting early corticosteroid use may improve outcomes if infection is controlled [4]. The use of multiple ophthalmic preparations (prednisolone, tobramycin, CMC, azithromycin) represents a meticulous approach to ocular protection, a key determinant of long-term morbidity often underemphasized in routine care [11]. One of the unique aspects was the comprehensive topical and systemic regimen involving 22 different interventions, including zinc-containing scalp lotion, triple-antibiotic powders, and Aloe-Vitamin E moisturizers. While these are not standard components of SJS-TEN treatment protocols, their use here reflects a holistic approach to wound healing, pain management, and prevention of superinfection, warranting further investigation for potential inclusion in broader care guidelines. A particularly rare feature in this case was the extensive involvement of nasal mucosa with crusting, which is barely described in the literature. Most reports focus on oral, ocular, and genital mucosa, with little emphasis on nasal damage. This suggests that nasal mucosal injury may be underrecognized or underreported in SJS-TEN. Additionally, the co-occurrence of burning micturition and reduced urine output with only trace hematuria and ketonuria may indicate early renal tubular involvement. Another unique point is the absence of a single clearly implicated drug, instead pointing to a polypharmacy-induced SJS-TEN, which is less frequently highlighted in case reports but increasingly relevant in modern prescribing contexts. It underscores the need for evaluating drug-drug interactions and cumulative immunogenicity in hypersensitivity reactions.

### Limitations

The diagnosis of Stevens-Johnson Syndrome (SJS) and Toxic Epidermal Necrolysis (TEN) in this case was primarily based on clinical features and temporal association with suspected drugs (ofloxacin, paracetamol, and cefixime), without histopathological confirmation due to the unavailability of skin biopsy. Additionally, immunological assays such as the drug-induced lymphocyte stimulation test (DLST) were not performed, limiting the ability to definitively identify the causative agent or assess potential drug–drug interactions. These diagnostic constraints represent significant limitations, which are acknowledged and considered in the interpretation of the findings and conclusions.

## Conclusion

This case highlights a severe SJS-TEN overlap likely triggered by multiple concurrently administered drugs, including ofloxacin, cefixime, and paracetamol. While polypharmacy appears to have contributed to the severity of the reaction, a definitive drug–drug interaction as the direct cause cannot be confirmed. The temporal sequence of drug exposure and clinical onset supports the involvement of these agents, consistent with previously reported cases in the literature. However, in the absence of confirmatory immunological testing or rechallenge, the exact causative mechanism remains uncertain. Therefore, conclusions are drawn with due clinical reasoning and cautious interpretation, emphasizing the need for careful drug prescription and monitoring, especially in patients receiving multiple medications.

## Data Availability

No datasets were generated or analysed during the current study.

## Abbreviations

APC: Antigen-presenting cell
BD: Bis in die (twice a day)
BSA: Body surface area
BUN: Blood urea nitrogen
CD4: Cluster of differentiation 4
CD8: Cluster of differentiation 8
CRISTEN: Clinical Risk Score for Toxic Epidermal Necrolysis
CYP2C19: Cytochrome P450 Family 2 Subfamily C Member 19
CYP2C9: Cytochrome P450 Family 2 Subfamily C Member 9
DIF: Direct Immunofluorescence
ELISA: Enzyme-Linked Immunosorbent Assay
EMM: Erythema Multiforme Major
FPR1: Formyl Peptide Receptor 1
HLA: Human Leukocyte Antigen
HLA: B15:02-Human Leukocyte Antigen B*15:02 Allele
HLA: B*57:01-Human Leukocyte Antigen B*57:01 Allele
HLA: E-Human Leukocyte Antigen E
IV: Intravenous
IVIG: Intravenous Immunoglobulin
MHC: Major Histocompatibility Complex
NA: Not Applicable
NETosis: Neutrophil Extracellular Trap-mediated Cell Death
NK cells: Natural Killer Cells
NSAIDs: Nonsteroidal Anti-inflammatory Drugs
OD: Once Daily
RDW: Red Cell Distribution Width
RHR: Red Blood Cell Distribution Width to Hemoglobin Ratio
SCARS: Severe Cutaneous Adverse Reactions
SCORTEN: Score for Toxic Epidermal Necrolysis
SJS: Stevens-Johnson Syndrome
SJS: TEN overlap-Stevens-Johnson Syndrome-Toxic Epidermal Necrolysis Overlap
TCR: T Cell Receptor
TEN: Toxic Epidermal Necrolysis
TNF: α-Tumor Necrosis Factor-alpha
TRAIL: TNF-Related Apoptosis-Inducing Ligand
Treg: Regulatory T Cells, w/v-Weight/Volume, w/w-Weight/Weight
TRM: Tissue-Resident Memory (T Cells)
